# The Effect of the Stress Induced by Hydrogen Peroxide and Corticosterone on Tryptophan Metabolism, Using Human Neuroblastoma Cell Line (SH-SY5Y)

**DOI:** 10.3390/ijms24054389

**Published:** 2023-02-23

**Authors:** Ana Salomé Correia, Isabel Silva, Henrique Reguengo, José Carlos Oliveira, Francisco Vasques-Nóvoa, Armando Cardoso, Nuno Vale

**Affiliations:** 1 OncoPharma Research Group, Center for Health Technology and Services Research (CINTESIS), Rua Doutor Plácido da Costa, 4200-450 Porto, Portugal; 2 Institute of Biomedical Sciences Abel Salazar (ICBAS), University of Porto, Rua de Jorge Viterbo Ferreira 228, 4050-313 Porto, Portugal; 3 CINTESIS@RISE, Faculty of Medicine, University of Porto, Alameda Professor Hernâni Monteiro, 4200-319 Porto, Portugal; 4 Clinical Chemistry, Department of Laboratory Pathology, Hospital Center of the University of Porto (CHUP), Largo Prof. Abel Salazar, 4099-313 Porto, Portugal; 5 Unit for Multidisciplinary Research in Biomedicine (UMIB), University of Porto, Rua de Jorge Viterbo Ferreira 228, 4050-313 Porto, Portugal; 6 Cardiovascular R and D Center, Faculty of Medicine, University of Porto, Rua Doutor Plácido da Costa, s/n, 4200-450 Porto, Portugal; 7 Department of Surgery and Physiology, Faculty of Medicine, University of Porto, Rua Doutor Plácido da Costa, 4200-450 Porto, Portugal; 8 NeuroGen Research Group, Center for Health Technology and Services Research (CINTESIS), Rua Doutor Plácido da Costa, 4200-450 Porto, Portugal; 9 Unit of Anatomy, Department of Biomedicine, Faculty of Medicine, University of Porto, Alameda Professor Hernâni Monteiro, 4200-319 Porto, Portugal; 10 Department of Community Medicine, Health Information and Decision (MEDCIDS), Faculty of Medicine, University of Porto, Rua Doutor Plácido da Costa, 4200-450 Porto, Portugal

**Keywords:** oxidative stress, glucocorticoids, tryptophan, 5-hydroxytryptophan, serotonin, 5-hydroxyindoleacetic acid

## Abstract

L-tryptophan (L-Trp) is an important amino acid in several physiological mechanisms, being metabolized into two important pathways: the kynurenine and the serotonin (5-HT) pathways. It is important in processes such as mood and stress response, the 5-HT pathway begins with the conversion of L-Trp to 5-hydroxytryptophan (5-HTP), that is metabolized into 5-HT, converted to melatonin or to 5-hydroxyindoleacetic acid (5-HIAA). Disturbances in this pathway are reported to be connected with oxidative stress and glucocorticoid-induced stress, are important to explore. Thus, our study aimed to understand the role of hydrogen peroxide (H_2_O_2_) and corticosterone (CORT)-induced stress on the serotonergic pathway of L-Trp metabolism, and on SH-SY5Y cells, focusing on the study of L-Trp, 5-HTP, 5-HT, and 5-HIAA in combination with H_2_O_2_ or CORT. We evaluated the effect of these combinations on cellular viability, morphology, and on the extracellular levels of the metabolites. The data obtained highlighted the different ways that stress induction led to different extracellular medium concentration of the studied metabolites. These distinct chemical transformations did not lead to differences in cell morphology/viability. Additionally, serotonin may be the most sensitive metabolite to the exposure to the different stress inducers, being more promissory to study conditions associated with cellular stress.

## 1. Introduction

L-tryptophan (L-Trp) is an essential amino acid, and is important in several physiological mechanisms, such as gastrointestinal and nervous processes. The metabolism of this amino acid is divided mainly in two branches: the kynurenine and the serotonin (5-HT) pathways [1,2]. Nevertheless, the metabolism of this amino acid is complex. Indeed, other compounds may be formed in the metabolism of this compound by the cells, such as indole-3-acetonitrile [3].

The kynurenine pathway represents about 95% of the metabolism of L-Trp [1]. This pathway has a crucial role on processes, such as inflammation and immune response, being connected to diseases, such as depression, cancer, and diabetes [4,5]. The 5-HT pathway is also extremely relevant in several physiological mechanisms. Indeed, processes, such as mood, sexuality, memory, appetite, stress response, motor control, regulation of circadian rhythm, gastrointestinal regulation, nociception, regulation of heart rate, and platelet aggregation, are influenced by this pathway [6]. Focusing on the 5-HT pathway, L-Trp is converted to 5-hydroxytryptophan (5-HTP) by tryptophan hydroxylase 1 or 2 (TPH1 or 2), the rate-limiting step. 5-HTP forms 5-HT by decarboxylation carried out by aromatic acid decarboxylase (AADC). Then, 5-HT may be converted to melatonin by metabolization to N-acetylserotonin (NAS), or to form 5-hydroxyindoleacetic acid (5-HIAA), the major 5-HT metabolite, by the enzyme monoamine oxidase (MAO) [7]. 

The impairment of L-Trp/5-HT pathways and oxidative stress and glucocorticoid-induced stress is important to explore, being already reported in some studies [8,9,10,11,12]. Indeed, oxidative stress generates neurotoxic compounds by the oxidation of the L-Trp. It also known that L-Trp may be metabolized to kynurenine directly by high levels of reactive oxygen species (ROS). Then, kynurenine may be metabolized to the oxidant compounds 3-hydroxykynurenine and quinolinic acid, associated with diseases, such as depressive disorders [13,14]. A previous study also revealed that L-Trp counteracted the cellular stress induced by hydrogen peroxide (H_2_O_2_), highlighting the role of L-Trp metabolism in the context of oxidative stress [8,15]. High levels of glucocorticoids are also reported to increase the kynurenine production, decreasing the activity of the 5-HT branch of L-Trp metabolism [12]. In patients with suicidal attempts, high levels of cortisol correlate with lower levels of L-Trp in the plasma, and a higher kynurenine/L-Trp ratio were also previously reported [16]. The administration of crocin also reduced cortisol levels and increased 5-HT in mice [17], supporting the inverse relationship between the levels of glucocorticoids and 5-HT.

Thus, our study aimed to explore the role of H_2_O_2_ and corticosterone (CORT)-induced stress on the L-Trp/5-HT metabolism, on SH-SY5Y cells previously used [8,9,18]. These cells are originally derived from a metastatic bone tumor biopsy, being widely used in the research of neuropsychiatric and neurological disorders and mechanisms, mainly because they have several neuronal features. Indeed, these cells can have cholinergic, adrenergic, or dopaminergic phenotypes, being frequently used in the study of Parkinson’s disease and depressive disorders [19,20]. It is also reported that SH-SY5Y cells produce 5-HT and express major serotonergic proteins, including the serotonergic type 1A receptor (5-HT_1A_) [21]. H_2_O_2_ induces direct oxidative stress, whereas high levels of CORT mimic the stress induced by hypothalamus-pituitary-adrenal axis disturbances [8,9]. 

**Figure 1:**
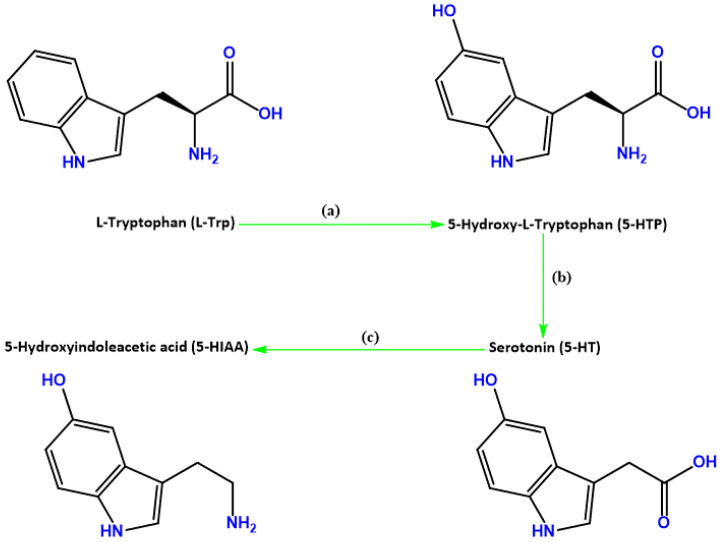
Summary of serotonin (5-HT)’s branch of L-tryptophan (L-Trp) biotransformation. This amino acid is (**a**) metabolized into 5-hydroxytryptophan (5-HTP), that is (**b**) converted into 5-HT, and finally (**c**) metabolized into 5-hydroxyindoleacetic acid (5-HIAA).

Thus, to study this induced stress on L-Trp metabolism, we focused on the study of L-Trp, 5-HTP, 5-HT, and 5-HIAA (Figure 1). These compounds were added in high concentrations to the cells, either alone or in combination with H_2_O_2_ or CORT aiming to evaluate the effect on cellular viability, morphology, and on the extracellular levels of the studied metabolites. 

Our results highlighted that different ways of stress induction led to different extracellular medium concentration of L-Trp, 5-HTP, 5-HT, and 5-HIAA. We also observed that these different chemical transformations did not lead to differences in cell morphology/viability between these different metabolites. Additionally, we hypothesized that the 5-HT pathway is the most sensitive and suitable to modulating effects of the cellular environment, and, therefore, presents the best applicability to be used in conditions associated with cellular stress. 

The most important abbreviations used in this manuscript are present on Table S1.

## 2. Results

### 2.1. Effect of H_2_O_2_ and CORT on SH-SY5Y Cellular Viability

**Figure 2:**
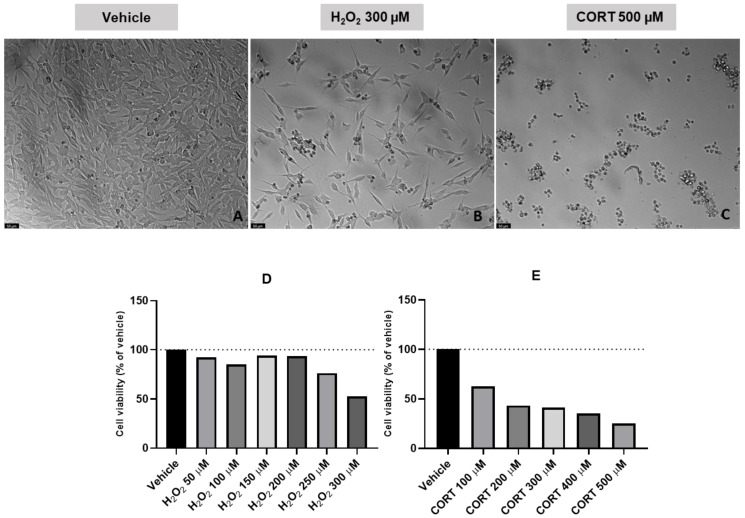
Representative images (100 × total magnification) of SH-SY5Y cells after incubation with (**A**) vehicle (H_2_O 1%), (**B**) hydrogen peroxide (H_2_O_2_) 300 µM, (**C**) corticosterone (CORT) 500 µM, and the effect of (**D**) H_2_O_2_ 50 µM–300 µM and (**E**) CORT 100 µM–500 µM on cellular viability. All the compounds were exposed to the cells for 48 h. Cell viability results are expressed as the percentage of the vehicle (100%). Scale bar: 50 μm.

In our previous works, we determined the effect of crescent concentrations of H_2_O_2_ and CORT on the viability of SH-SY5Y cells [8,9]. Just to confirm these results, we repeated this procedure from one independent culture preparation, adding H_2_O_2_ 50–300 µM and CORT 100–500 µM to the cells for 48 h. After that, we evaluated the effect of these compounds on the morphology and viability of SH-SY5Y cells. Figure 2 represents the obtained results.

These results confirm that both H_2_O_2_ and CORT decreased cellular viability in a concentration-dependent manner. Cellular viability values were 52.84% and 25.05% with H_2_O_2_ 300 µM and CORT 500 µM, respectively. Additionally, cell viability values are also concordant with the cellular morphology. Imagens demonstrate cell damage after application of H_2_O_2_ 300 µM and CORT 500 µM, reflected by rounded morphology, shrinkage, and, overall, a lower number of cells, typical features of cell death [22].

### 2.2. Effect of L-Trp and Combinations of L-Trp with H_2_O_2_ or CORT on SH-SY5Y Cell Viability and Extracellular L-Trp Concentration 

**Figure 3:**
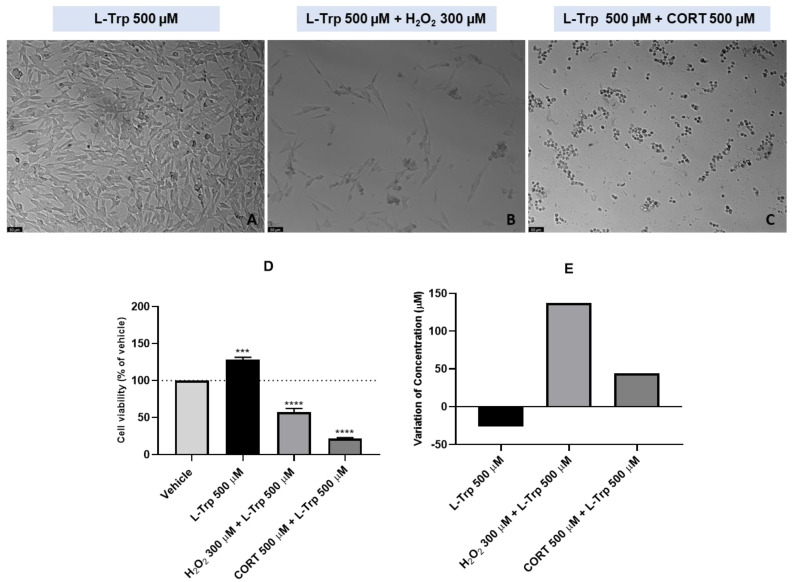
Representative images (100 × total magnification) of SH-SY5Y cells exposed to (**A**) L-Trp 500 µM, (**B**) L-Trp 500 µM + H_2_O_2_ 300 µM, (**C**) L-Trp 500 µM + CORT 500 µM, and the effect of L-Trp 500 µM, L-Trp 500 µM + H_2_O_2_ 300 µM, L-Trp 500 µM + CORT 500 µM on (**D**) cellular viability, and on (**E**) the variation of L-Trp concentration, in which the baseline represents L-Trp 500 µM. All the compounds were exposed to the cells for 48 h. Cell viability results represent the mean ± SEM of three independent assays, expressed as the percentage of the vehicle (100%). HPLC results represent the analysis of the mixture of samples from three independent experiments. Statistically significant *** *p* < 0.001 and **** *p* < 0.0001 vs. vehicle. Scale bar: 50 µm.

To evaluate the 48 h effect of L-Trp and combinations of this amino acid with H_2_O_2_ or CORT on SH-SY5Y cellular viability and extracellular L-Trp concentration, this compound was added to the cells in a concentration of 500 µM. In turn, H_2_O_2_ and CORT were added to the cells in a concentration of 300 µM and 500 µM, respectively. As mentioned above, the purpose of these high concentrations was to ensure that we could obtain a measurable response of the cells to these compounds, allowing a better interpretation and examination of the results, mainly on the HPLC. After exposing the cells to the different treatment conditions, we evaluated the effect on the morphology and viability of SH-SY5Y cells (Figure 3A–D). The extracellular media was also collected, aiming to understand the effect of these treatments on the L-Trp extracellular concentration (Figure 3E).

Our results revealed that L-Trp (single compound) increased cell viability (128.39% vs. vehicle). On the other hand, when combined with both H_2_O_2_ and CORT, cell viability decreased (57.12% vs. vehicle and 21.55% vs. vehicle, respectively). However, analyzing HPLC results, we observed the opposite. Indeed, after the 48 h exposure of the cells to L-Trp (single compound), the concentration of L-Trp in the extracellular medium decreased 26 µM. When combined with both H_2_O_2_ and CORT, L-Trp levels in the extracellular medium increased 137 µM and 44 µM, respectively. Viability values and morphology assessment were also concordant. Indeed, lower viability values were reflected in rounded morphology, shrinkage, and a lower number of cells.

### 2.3. Effect of 5-HTP and Combinations of 5-HTP with H_2_O_2_ or CORT on SH-SY5Y Cell Viability and Extracellular 5-HTP Concentration

**Figure 4:**
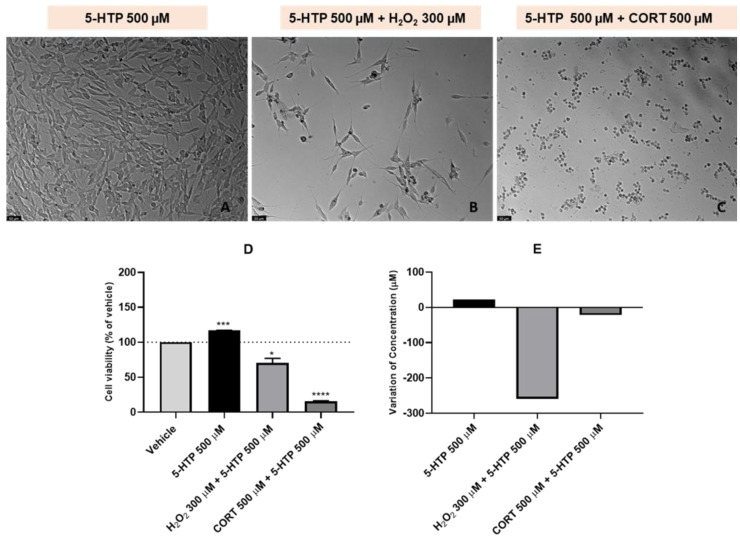
Representative images (100× total magnification) of SH-SY5Y cells exposed to (**A**) 5-HTP 500 µM, (**B**) 5-HTP 500 µM + H_2_O_2_ 300 µM, (**C**) 5-HTP 500 µM + CORT 500 µM, and effect of 5-HTP 500 µM, 5-HTP 500 µM + H_2_O_2_ 300 µM, 5-HTP 500 µM + CORT 500 µM on (**D**) cellular viability and on (**E**) the variation of 5-HTP concentration, in which the baseline represents 5-HTP 500 μM. All the compounds were exposed to the cells for 48 h. Cell viability results represent the mean ± SEM of three independent assays, expressed as the percentage of the vehicle (100%). HPLC results represent the analysis of the mixture of samples from three independent experiments. Statistically significant * *p* < 0.05, *** *p* < 0.001 and **** *p* < 0.0001 vs. vehicle. Scale bar: 50 μm.

To evaluate the 48 h effect of 5-HTP and combinations of this compound with H_2_O_2_ or CORT on the viability of SH-SY5Y cells and on extracellular 5-HTP concentration, this compound was added to the cells in a concentration of 500 µM. H_2_O_2_ and CORT were added to the cells in a concentration of 300 µM and 500 µM, respectively. After exposing the cells to the different treatment conditions, we assessed the effect on the morphology and viability of SH-SY5Y cells (Figure 4A–D). The extracellular media was also collected, with the aim to understand the effect of these treatments on the 5-HTP extracellular concentration (Figure 4E).

Our data demonstrated that, similar to what happened with L-Trp, 5-HTP single compound increased cell viability (116.81% vs. vehicle). When combined with H_2_O_2_ and CORT, cell viability decreased (70.51% vs. vehicle and 15.76% vs. vehicle, respectively). HPLC results revealed that after the 48 h exposition of the cells to 5-HTP alone, the concentration of this compound in the extracellular medium slightly increased (22 µM). When combined with both H_2_O_2_ and CORT, 5-HTP levels in the extracellular medium decreased 259 µM and 22 µM, respectively, following an opposite tendency compared to the L-Trp levels on the extracellular medium. Regarding morphological evaluation, once again, these images supported viability values.

### 2.4. Effect of 5-HT and Combinations of 5-HT with H_2_O_2_ or CORT on SH-SY5Y Cell Viability and Extracellular 5-HT Concentration

**Figure 5:**
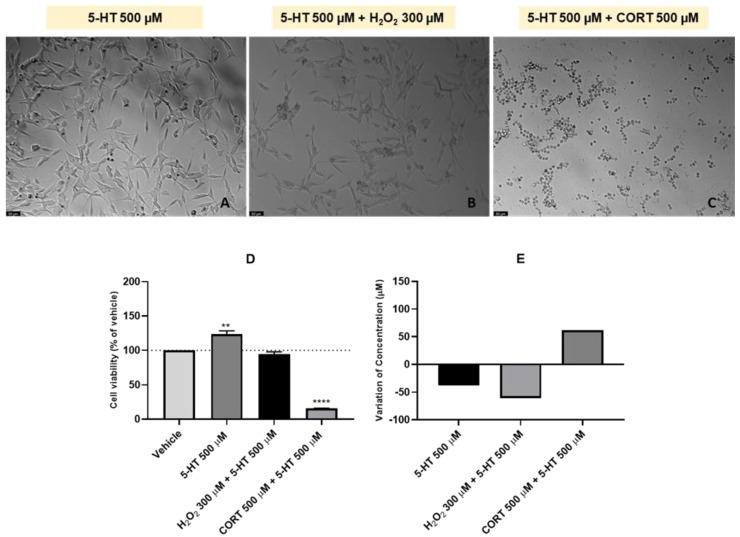
Representative images (100× total magnification) of SH-SY5Y cells exposed to (**A**) 5-HT 500 µM, (**B**) 5-HT 500 µM + H_2_O_2_ 300 µM, (**C**) 5-HT 500 µM + CORT 500 µM, and effect of 5-HT 500 µM, 5-HT 500 µM + H_2_O_2_ 300 µM, 5-HT 500 µM + CORT 500 µM on (**D**) cellular viability, and on (**E**) the variation of 5-HT concentration, in which the baseline represents 5-HT 500 µM. All the compounds were exposed to the cells for 48 h. Cell viability results represent the mean ± SEM of three independent assays, expressed as the percentage of the vehicle (100%). HPLC results represent the analysis of the mixture of samples from three independent experiments. Statistically significant ** *p* < 0.01 and **** *p* < 0.0001 vs. vehicle. Scale bar: 50 µm.

Continuing the study of L-Trp metabolic cascade, to study the 48 h effect of 5-HT and combinations of this compound with H2O2 or CORT on the viability of SH-SY5Y cells and on extracellular 5-HT concentration, this compound was also added to the cells in a concentration of 500 µM. H_2_O_2_ and CORT were added to the cells in a concentration of 300 µM and 500 µM, respectively. After exposing the cells to the different treatment conditions, we assessed the effect on the morphology and viability of SH-SY5Y cells (Figure 5A–D). Once again, the extracellular media was collected, with the aim to understand the effect of these treatments on the 5-HT extracellular concentration (Figure 5E).

The obtained results evidence that 5-HT isolated slightly increased cell viability (123.74% vs. vehicle). On the other hand, when combined with both stress inducers, decreased cell viability. Combined with H_2_O_2_, the cellular viability was only slightly different than the vehicle (94.21%). However, combined with CORT, cell viability decreased sharply (15.89% vs. vehicle). Regarding the HPLC results, after the 48 h exposure of the SH-SY5Y cells to 5-HTP alone, the concentration of this compound in the extracellular medium decreased (38 µM). However, and in a different way than what was observed with the other analyzed compounds, when 5-HT was combined with both H_2_O_2_ and CORT, an opposite tendency in the extracellular levels of this neurotransmitter was observed. Indeed, when combined with H_2_O_2,_ 5-HT levels in the extracellular medium decreased (61 µM), and when combined with CORT, these values increased (62 µM). It is important to note that viability and morphology assessment are in concordance.

### 2.5. Effect of 5-HIAA and Combinations of 5-HIAA with H_2_O_2_ or CORT on SH-SY5Y Cell Viability and Extracellular 5-HIAA Concentration

**Figure 6:**
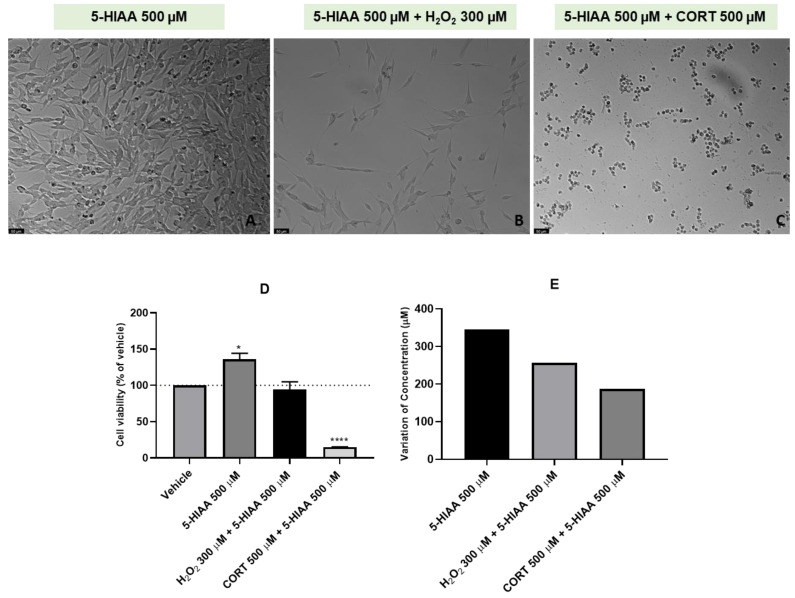
Representative images (100 × total magnification) of SH-SY5Y cells exposed to (**A**) 5-HIAA 500 µM, (**B**) 5-HIAA 500 µM + H_2_O_2_ 300 µM, (**C**) 5-HIAA 500 µM + CORT 500 µM, and effect of 5-HIAA 500 µM, 5-HIAA 500 µM + H_2_O_2_ 300 µM, 5-HIAA 500 µM + CORT 500 µM on (**D**) cellular viability, and on (**E**) the variation of 5-HIAA concentration, in which the baseline represents 5-HIAA 500 μM. All the compounds were exposed to the cells for 48 h. Cell viability results represent the mean ± SEM of three independent assays, expressed as the percentage of the vehicle (100%). HPLC results represent the analysis of the mixture of samples from three independent experiments. Statistically significant * *p* < 0.05, and **** *p* < 0.0001 vs. vehicle. Scale bar: 50 μm.

Finally, to study the 48 h effect of 5-HIAA and combinations of this compound with H_2_O_2_ or CORT on the viability of SH-SY5Y cells and on extracellular 5-HIAA concentration, this compound was also added to the cells in a concentration of 500 µM. Again, H_2_O_2_ and CORT were added to the cells in a concentration of 300 µM and 500 µM, respectively. After exposing the cells to the different treatment conditions, we evaluated the effect on the morphology and viability of SH-SY5Y cells (Figure 6A–D). The extracellular media was also collected, aiming to understand the effect of these treatments on the extracellular concentration of 5-HIAA (Figure 6E). 

Our results revealed that 5-HIAA (alone) increased cell viability (136.06% vs. vehicle). On the other hand, when combined with both H_2_O_2_ and CORT, cell viability decreased slightly with H_2_O_2_ (94.28% vs. vehicle) and decreased markedly with CORT (15.07% vs. vehicle). Analyzing HPLC results, we observed marked increase in the 5-HIAA extracellular levels after all the treatments. Indeed, after the 48 h exposure of the cells to 5-HIAA (single compound), the concentration of this compound in the extracellular medium increased 345 µM. When combined with both H_2_O_2_ and CORT, L-Trp levels in the extracellular medium increased 256 µM and 187 µM, respectively. Viability values and morphology assessment were also concordant. In fact, lower viability values were reflected in rounded morphology, shrinkage, and a lower number of cells.

### 2.6. Effect of H_2_O_2_ and CORT on Extracellular 5-HT Concentration

**Figure 7:**
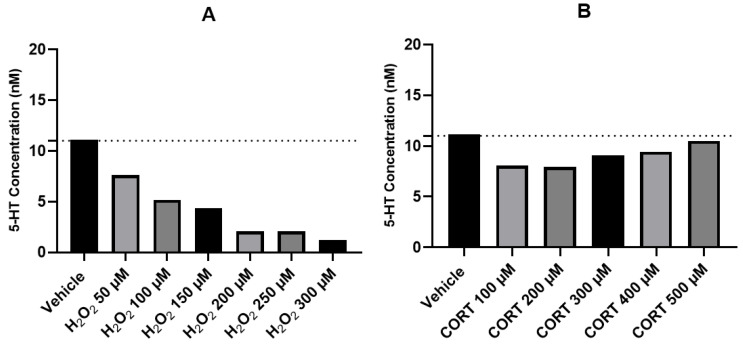
Concentrations of 5-HT (nM) in the extracellular medium of SH-SY5Y cells treated with (**A**) H_2_O_2_ 50–300 µM and (**B**) CORT 100–500 µM, determined by HPLC (electrochemical method). The results represent the analysis of the supernatant collected from the three independent experiments.

Based on the results obtained with 5-HT and the importance of this neurotransmitter on the study of neuropsychiatric diseases, we added H_2_O_2_ 50–300 µM and CORT 100–500 µM to cells, for 48 h, aiming to understand the effect of these treatments on the extracellular concentration of 5-HT. In this study, we used an electrochemical method of HPLC, more sensitive to detect 5-HT concentrations produced by the cells. Figure 7 represents the obtained results.

These results demonstrate that after exposing SH-SY5Y cells for 48 h with crescent concentrations of H_2_O_2,_ 5-HT levels on the extracellular medium decreased in a concentration-dependent way, varying between 11.12 nM (vehicle) and 1.19 nM (H_2_O_2_ 300 µM). Regarding CORT, the exposition of this compound to the cells did not significantly alter 5-HT concentrations in the extracellular medium. Indeed, CORT 100 μM led to 5-HT values of 8.05 nM. This value increased with the concentration of CORT, being near the vehicle value (11.12 nM) with CORT 500 µM (10.51 nM).

## 3. Discussion

L-Trp is an important amino acid in numerous physiological mechanisms, being the precursor of 5-HT synthesis [1]. The role of oxidative stress and glucocorticoid-induced stress in the impairment of the serotonergic pathway of L-Trp metabolism is important to explore, being the aim of our study. The data obtained with the application of H_2_O_2_ and CORT to the cells is consistent with previous studies [8,9,23,24], despite some limitations with the use of these agents. Particularly, H_2_O_2_ is an unstable compound, rapidly decomposing to oxygen and water, demanding a careful handle [25]. It is also important to note that cortisol is the primary endogenous adrenal steroid in humans, whereas CORT is the primary adrenal corticosteroid in rodents [26]. However, both glucocorticoids have a high level of similarity, being widely reported the use of CORT on human cells [27,28]. Indeed, previously, we also tested the effect of the synthetic form of cortisol (hydrocortisone), obtaining no responses regarding cellular viability, morphology, or DNA damage [9].

Nevertheless, despite limitations, it is widely reported that these agents induce oxidative stress, known to trigger processes, such as inflammation, neurodegeneration, tissue damage and cell death, particularly relevant for depressive disorders [29]. Based in our data, we selected H_2_O_2_ 300 µM and CORT 500 µM to proceed HPLC analyses, aiming to ensure that we could obtain a measurable response of the cells to these compounds, allowing a better interpretation and examination of the results. In this study, the combination of these agents with L-Trp, 5-HTP, 5-HT, and 5-HIAA did not alleviate the harmful effect of both stressors, demonstrating that these high concentrations of CORT and H_2_O_2_ influenced the observed cellular viability inducer effect of the isolated L-Trp metabolites. In our previous studies, we revealed that L-Trp and mirtazapine (an antidepressant that act on serotonergic-related pathways) alleviated cellular stress, demonstrating the importance of this pathway for cellular viability, and recovery from the harmful effects of high levels of oxidative stress [8,9]. Indeed, several studies demonstrate the importance of compounds that act on L-Trp/5-HT pathway in the relieve of glucocorticoid and oxidative stress [10,30,31,32]. 

After a period of 48 h, the extracellular concentration of each added metabolite markedly varied, independently of cellular viability values. In fact, the obtained values regarding cellular viability and morphology were similar among all metabolites. This did not happen with the results obtained through the determination of the concentration in the extracellular medium demonstrating that different chemical transformations occurred which, during the study period, did not lead to differences in cell morphology/viability.

It is particularly noteworthy that regarding cellular viability and morphology, the results obtained with the combination of each metabolite with CORT revealed greater cell damage than with H_2_O_2_. However, the results obtained through the determination of the concentration in the extracellular medium clearly demonstrated that there was a much more pronounced variation with the combinations of the metabolites with H_2_O_2_ than with CORT, which can be explained by the high oxidative character of the H_2_O_2_ [33]. Indeed, previously, we also demonstrated that regarding ROS production, overall, CORT led to low levels of production compared to H_2_O_2_ [8]. This highlights that the way of inducing stress in cells leads to different responses, being an important future topic of studies.

It is important to note that L-Trp, 5-HTP, and 5-HIAA had the same tendency to decrease/increase extracellular concentration when combined with both CORT and H_2_O_2_. Regarding 5-HT, this trend was opposite between both stress inducer agents, revealing an increase in the extracellular concentration of this neurotransmitter when combined with CORT and a decrease when combined with H_2_O_2_. The results obtained with the electrochemical method for the detection of 5-HT, precisely demonstrated this trend regarding to the extracellular levels of 5-HT only with the addition of CORT/ H_2_O_2_. These results may indicate that different forms of stress induction affect 5-HT levels differently, mainly in short periods of time. Indeed, 5-HT has a known protective effect on oxidation processes because it protects membrane lipids from oxidation [34]. Possibly this compound acts on the oxidative effect of H_2_O_2_, being more consumed. This antioxidant power of 5-HT makes this pathway attractive for the focus on the development of drugs aiming to interact with serotonergic receptors in the context of stress.

Regarding both L-Trp and 5-HIAA, it can be observed that there was an increase in their extracellular levels after being combined with CORT and especially with H_2_O_2_. In fact, it is known that L-Trp is easily oxidized by free radicals, leading to products, such as tryptophanyl (indolyl) radical (Trp) [35], easily masked with L-Trp in its non-oxidized form. Concerning to 5-HIAA, the very high extracellular concentration values are possibly due to the ease of conjugation of this metabolite with other compounds. For example, it is known that urine samples need to be hydrolyzed to release 5-HIAA from conjugates, demonstrating that this compound is easily conjugated with other compounds [36]. With 5-HTP, there is a decrease in the levels of this metabolite. Indeed, its hydroxyl group makes it highly susceptible to oxidation, especially by H_2_O_2_ [37]. 

Studying extracellular levels of L-Trp, 5-HTP, 5-HT, and 5-HIAA after exposition to different stress stimuli is also important to ensure the fine equilibrium between low/high levels of these metabolites and homeostatic levels, that may be maintained. For example, regarding 5-HT, low levels of this neurotransmitter are associated with several pathologies, such as depressive disorders [38]. High levels of this neurotransmitter are also associated with pathological conditions, such as the serotonin syndrome, a potentially life-threatening disease [39].

## 4. Materials and Methods

### 4.1. Materials

Dulbecco’s Modified Eagle’s Medium (DMEM, cat. no. FG0415) and Fetal Bovine Serum (FBS, cat. no. S0615) were purchased from Millipore Sigma (Merck KGaA, Darmstadt, Germany). Penicillin/streptomycin (cat. no. P4333), thiazolyl blue tetrazolium bromide (MTT, cat. no. M5655), CORT (cat. no. 27840), H_2_O_2_ 30% (cat. no. 1.07209), L-Trp (cat. no. T0254-5G), 5-HTP (cat. no. H9772-1G), 5-HT (cat. no. H9523-25MG), and 5-HIAA (cat. no. H8876-100MG) were acquired from Sigma-Aldrich (Merck KGaA, Darmstadt, Germany).

For the HPLC procedure (electrochemical method), materials were previously described [40]. Ultra-high-performance liquid chromatograph (uHPLC; Flexar FX-10 Ultra High-Performance LC 10,000 PSI) was obtained from Perkin Elmer (Waltham, MA, USA). The column (C18 EQV-8986, 75 × 3.0 mm^2^ id) was obtained from ACE ^®^ (Aberdeen, UK). 

### 4.2. Cell Treatments

All the compounds, except CORT, were dissolved in sterilized water, maximum 1% in culture medium. CORT was dissolved in methanol, 0.1% in culture medium. L-Trp, 5-HT, 5-HTP, 5-HIAA, and CORT were added to the cells in a concentration of 500 µM. H_2_O_2_ was added to the cells in a concentration of 300 µM. Only for the performance of the electrochemical method of HPLC, CORT, and H_2_O_2_ were added to the cells in concentrations ranging 100–500 µM and 50–300 µM, respectively. All the compounds were added to the cells for 48 h.

### 4.3. Cell Culture

SH-SY5Y cell line (American Type Culture Collection, VA, USA) was cultured in DMEM (10% FBS, 1% penicillin (1000 U/mL)/streptomycin (10 mg/mL)), and incubated at 37 °C (5% CO_2_). Cells were seeded at a density of 1.0 × 10^5^ cells/mL in 96-well plates (200 µL/well), after trypsinization (0.25% trypsin-EDTA), and centrifugation (1100 rpm for SH-SY5Y, 5 min; Hettich, Tuttlingen, Germany).

### 4.4. Thiazolyl Blue Tetrazolium Bromide Assay

Cell viability was obtained by thiazolyl blue tetrazolium bromide (MTT) assay, after 48 h of exposure to the compounds, as previously reported [8,9]. To perform that, the culture medium was removed, and MTT (0.5 mg/mL in PBS) was added to the cells (100 µL/well), following a 3 h period of incubation at 37 °C in a light protected manner. After that, MTT was discarded and DMSO (100 µL/well) was added to the cells. Finally, absorbance values (570 nm) were obtained with the automated microplate reader (Tecan Infinite M200, Zurich, Switzerland). 

### 4.5. Cell Morphology Assessment 

The cellular morphology of SH-SY5Y cell lines was assessed by using Leica DMI6000 B Automated Microscope (Wetzlar, Germany) after all the exposure to the different treatment conditions for 48 h.

### 4.6. HPLC Analysis

HPLC (electrochemical method) analysis was carried out as previously described [40]. Briefly, the analysis of 5-HT content in the samples was carried out using the 3030 Reagent kit for HPLC analysis of this compound in the urine. The calibration curve was generated with concentrations between 1–1000 nM of 5-HT (y = 493146*x*; R² *=* 0.9963). The decade electrochemical detector contained a glassy carbon electrode programmed to a potential of 50 mV. Empower Pro software 3 (Waters Corporation, Milford, MA, USA) was used for controlling the produced current For uHPLC procedure, samples (20 µL, 4 °C) were subjected to the uHPLC, and the separation was carried out at a flux of 2 mL/min. Optical density of all the tested compounds was recorded at 280 nm. Quantification was performed based on standard curves for L-Trp (y = 750,542*x*; R² = 0.9971), 5-HTP (y = 804,860*x*; R² = 0.9986), 5-HT (y = 782,455*x;* R² = 0.9977), and 5-HIAA (y = 595,569*x;* R² = 0.9919). Results were analyzed on using Chromera^®^ software, version 3.2.0, Perkin Elmer (Waltham, MA, USA).

### 4.7. Statistical and Data Analysis 

Statistical and data analysis was performed using the software GraphPad Prism 8 (San Diego, CA, USA). Statistical comparisons were performed between vehicle and treatment groups (one-way ANOVA and Dunnett’s multiple comparisons test), statistical significance when *p* < 0.05. Cell viability studies results represent the mean ± SEM of 3 independent experiments. HPLC results represent the analysis of the mixture of samples of three independent experiments.

## 5. Conclusions

Taken together, our results highlight that different ways of stress induction lead to different responses regarding SH-SY5Y extracellular medium concentration of L-Trp, 5-HTP, 5-HT, and 5-HIAA. Indeed, the different chemical transformations that occurred during the study period did not lead to differences in cell morphology/viability between the different metabolites. We believe that, based on the opposite results on the concentration of 5-HT in the extracellular medium after exposition to both CORT and H_2_O_2,_ the 5-HT pathway is the one that is most sensitive to modulating effects of the cellular environment and, therefore, presents the best applicability to be used in conditions associated with cellular stress. 

Future studies exploring L-Trp metabolism with a focus on 5-HT branch are important in the context of several diseases that are extremely influenced by stress conditions, such as depressive disorders [29,41].

## Data Availability

Not applicable.

## Supplementary Materials

The following supporting information can be downloaded at: https://www.mdpi.com/article/10.3390/ijms24054389/s1.

## References

[1] Platten M., Nollen E.A.A., Röhrig U.F., Fallarino F., Opitz C.A. (2019). Tryptophan metabolism as a common therapeutic target in cancer, neurodegeneration and beyond. Nat. Rev. Drug Discov..

[2] Correia A.S., Vale N. (2022). Tryptophan Metabolism in Depression: A Narrative Review with a Focus on Serotonin and Kynurenine Pathways. Int. J. Mol. Sci..

[3] Duarte D., Amaro F., Silva I., Silva D., Fresco P., Oliveira J.C., Reguengo H., Gonçalves J., Vale N. (2019). Carbidopa Alters Tryptophan Metabolism in Breast Cancer and Melanoma Cells Leading to the Formation of Indole-3-Acetonitrile, a Pro-Proliferative Metabolite. Biomolecules.

[4] Cervenka I., Agudelo L.Z., Ruas J.L. (2017). Kynurenines: Tryptophan’s metabolites in exercise, inflammation, and mental health. Science.

[5] Savitz J. (2020). The kynurenine pathway: A finger in every pie. Mol. Psychiatry.

[6] Berger M., Gray J.A., Roth B.L. (2009). The Expanded Biology of Serotonin. Annu. Rev. Med..

[7] Höglund E., Øverli Ø., Winberg S. (2019). Tryptophan Metabolic Pathways and Brain Serotonergic Activity: A Comparative Review. Front. Endocrinol..

[8] Correia A.S., Cardoso A., Vale N. (2022). Significant Differences in the Reversal of Cellular Stress Induced by Hydrogen Peroxide and Corticosterone by the Application of Mirtazapine or L-Tryptophan. Int. J. Transl. Med..

[9] Correia A.S., Fraga S., Teixeira J.P., Vale N. (2022). Cell Model of Depression: Reduction of Cell Stress with Mirtazapine. Int. J. Mol. Sci..

[10] Wauquier F., Boutin-Wittrant L., Pourtau L., Gaudout D., Moras B., Vignault A., Monchaux De Oliveira C., Gabaston J., Vaysse C., Bertrand K. (2022). Circulating Human Serum Metabolites Derived from the Intake of a Saffron Extract (Safr’InsideTM) Protect Neurons from Oxidative Stress: Consideration for Depressive Disorders. Nutrients.

[11] Omachi T., Matsuyama N., Hasegawa Y. (2023). Nacre extract from pearl oyster suppresses LPS-induced depression and anxiety. J. Funct. Foods.

[12] Oxenkrug G.F. (2010). Tryptophan kynurenine metabolism as a common mediator of genetic and environmental impacts in major depressive disorder: The serotonin hypothesis revisited 40 years later. Isr. J. Psychiatry Relat. Sci..

[13] Bakunina N., Pariante C.M., Zunszain P.A. (2015). Immune mechanisms linked to depression via oxidative stress and neuroprogression. Immunology.

[14] Müller A., Leichert L.I. (2012). Redox proteomics. Oxidative Stress Redox Regul..

[15] Balmus I.M., Ciobica A., Antioch I., Dobrin R., Timofte D. (2016). Oxidative Stress Implications in the Affective Disorders: Main Biomarkers, Animal Models Relevance, Genetic Perspectives, and Antioxidant Approaches. Oxid Med. Cell Longev..

[16] Messaoud A., Mensi R., Douki W., Neffati F., Najjar M.F., Gobbi G., Valtorta F., Gaha L., Comai S. (2019). Reduced peripheral availability of tryptophan and increased activation of the kynurenine pathway and cortisol correlate with major depression and suicide. World J. Biol. Psychiatry.

[17] Zhang F., Zhu X., Yu P., Sheng T., Wang Y., Ye Y. (2022). Crocin ameliorates depressive-like behaviors induced by chronic restraint stress via the NAMPT-NAD^+^-SIRT1 pathway in mice. Neurochem. Int..

[18] Silva D., Rocha R., Correia A.S., Mota B., Madeira M.D., Vale N., Cardoso A. (2022). Repurposed Edaravone, Metformin, and Perampanel as a Potential Treatment for Hypoxia–Ischemia Encephalopathy: An In Vitro Study. Biomedicines.

[19] Jantas D. (2016). Cell-Based Systems of Depression: An Overview. Herbal Medicine in Depression: Traditional Medicine to Innovative Drug Delivery.

[20] Kovalevich J., Langford D. (2013). Considerations for the Use of SH-SY5Y Neuroblastoma Cells in Neurobiology. Methods in Molecular Biology.

[21] Walory J., Mielczarek L., Jarończyk M., Koronkiewicz M., Kossakowski J., Bugno R., Bojarski A., Chilmonczyk Z. (2018). Oncotoxic Properties of Serotonin Transporter Inhibitors and 5-HT1A Receptor Ligands. Int. J. Mol. Sci..

[22] Saraste A., Pulkki K. (2000). Morphologic and biochemical hallmarks of apoptosis. Cardiovasc. Res..

[23] Lieberknecht V., Engel D., Rodrigues A.L.S., Gabilan N.H. (2020). Neuroprotective effects of mirtazapine and imipramine and their effect in pro- and anti-apoptotic gene expression in human neuroblastoma cells. Pharmacol. Rep..

[24] Zhou B., Tan J., Zhang C., Wu Y. (2018). Neuroprotective effect of polysaccharides from *Gastrodia elata* Blume against corticosterone-induced apoptosis in PC 12 cells via inhibition of the endoplasmic reticulum stress-mediated pathway. Mol. Med. Rep..

[25] Hydrogen Peroxide|Toxic Substances|Toxic Substance Portal|ATSDR. https://wwwn.cdc.gov/TSP/substances/ToxSubstance.aspx?toxid=55.

[26] Raff H. (2016). CORT, Cort, B, Corticosterone, and now Cortistatin: Enough Already!. Endocrinology.

[27] Yu Z., Kong D., Liang Y., Zhao X., Du G. (2021). Protective effects of VMY-2-95 on corticosterone-induced injuries in mice and cellular models. Acta Pharm. Sin. B.

[28] Yang G., Li J., Cai Y., Yang Z., Li R., Fu W. (2018). Glycyrrhizic Acid Alleviates 6-Hydroxydopamine and Corticosterone-Induced Neurotoxicity in SH-SY5Y Cells through Modulating Autophagy. Neurochem. Res..

[29] Bhatt S., Nagappa A.N., Patil C.R. (2020). Role of oxidative stress in depression. Drug Discov. Today.

[30] Correia A.S., Cardoso A., Vale N. (2023). Oxidative Stress in Depression: The Link with the Stress Response, Neuroinflammation, Serotonin, Neurogenesis and Synaptic Plasticity. Antioxidants.

[31] Wang H., Zhou X., Huang J., Mu N., Guo Z., Wen Q., Wang R., Chen S., Feng Z.-P., Zheng W. (2013). The role of Akt/FoxO3a in the protective effect of venlafaxine against corticosterone-induced cell death in PC12 cells. Psychopharmacology.

[32] Ribaudo G., Bortoli M., Witt C.E., Parke B., Mena S., Oselladore E., Zagotto G., Hashemi P., Orian L. (2022). ROS-Scavenging Selenofluoxetine Derivatives Inhibit In Vivo Serotonin Reuptake. ACS Omega.

[33] Abdollahi M., Hosseini A. (2014). Hydrogen Peroxide. Encyclopedia of Toxicology.

[34] Azouzi S., Santuz H., Morandat S., Pereira C., Côté F., Hermine O., El Kirat K., Colin Y., Le Van Kim C., Etchebest C. (2017). Antioxidant and Membrane Binding Properties of Serotonin Protect Lipids from Oxidation. Biophys. J..

[35] Fuentes-Lemus E., Dorta E., Escobar E., Aspée A., Pino E., Abasq M.L., Speisky H., Silva E., Lissi E., Davies M.J. (2016). Oxidation of free, peptide and protein tryptophan residues mediated by AAPH-derived free radicals: Role of alkoxyl and peroxyl radicals. RSC Adv..

[36] Becker A., Schalin-Jäntti C., Itkonen O. (2021). Comparison of Serum and Urinary 5-Hydroxyindoleacetic Acid as Biomarker for Neuroendocrine Neoplasms. J. Endocr. Soc..

[37] Wang X., Zhang L. (2018). Kinetic study of hydroxyl radical formation in a continuous hydroxyl generation system. RSC Adv..

[38] Otte C., Gold S.M., Penninx B.W., Pariante C.M., Etkin A., Fava M., Mohr D.C., Schatzberg A.F. (2016). Major depressive disorder. Nat. Rev. Dis. Prim..

[39] Scotton W.J., Hill L.J., Williams A.C., Barnes N.M. (2019). Serotonin Syndrome: Pathophysiology, Clinical Features, Management, and Potential Future Directions. Int. J. Tryptophan Res..

[40] Correia A.S., Duarte D., Silva I., Reguengo H., Oliveira J.C., Vale N. (2021). Serotonin after β-Adrenoreceptors’ Exposition: New Approaches for Personalized Data in Breast Cancer Cells. J. Pers. Med..

[41] Russell G., Lightman S. (2019). The human stress response. Nat. Rev. Endocrinol..

