# Supplementary material for: The Effect of the Stress Induced by Hydrogen Peroxide and Corticosterone on Tryptophan Metabolism, Using Human Neuroblastoma Cell Line (SH-SY5Y)
Source: Int J Mol Sci. 2023 Feb 23;24(5):4389. doi: 10.3390/ijms24054389 (PMC10001894; doi:10.3390/ijms24054389)
Supplement: Supplementary file 1 [file ijms-24-04389-s001.zip › ijms-2244904-supplementary.pdf]

International Journal of *Molecular Sciences*

Article

The Effect of the Stress Induced by Hydrogen Peroxide and Corticosterone on Tryptophan Metabolism, Using Human Neuroblastoma Cell Line (SH-SY5Y)

Ana Salomé Correia [1,2,3], Isabel Silva [4], Henrique Reguengo [4,5], José Carlos Oliveira [2,4,5], Francisco Vasques-Nóvoa [6,7], Armando Cardoso [3,8,9] and Nuno Vale [1,3,10,*]

[1] OncoPharma Research Group, Center for Health Technology and Services Research (CINTESIS), Rua Doutor Plácido da Costa, 4200-450 Porto, Portugal
[2] Institute of Biomedical Sciences Abel Salazar (ICBAS), University of Porto, Rua de Jorge Viterbo Ferreira 228, 4050-313 Porto, Portugal
[3] CINTESIS@RISE, Faculty of Medicine, University of Porto, Alameda Professor Hernâni Monteiro, 4200-319 Porto, Portugal
[4] Clinical Chemistry, Department of Laboratory Pathology, Hospital Center of the University of Porto (CHUP), Largo Prof. Abel Salazar, 4099-313 Porto, Portugal
[5] Unit for Multidisciplinary Research in Biomedicine (UMIB), University of Porto, Rua de Jorge Viterbo Ferreira 228, 4050-313 Porto, Portugal
[6] Cardiovascular R&D Center, Faculty of Medicine, University of Porto, Rua Doutor Plácido da Costa, s/n, 4200-450 Porto, Portugal
[7] Department of Surgery and Physiology, Faculty of Medicine, University of Porto, Rua Doutor Plácido da Costa, 4200-450 Porto, Portugal
[8] NeuroGen Research Group, Center for Health Technology and Services Research (CINTESIS), Rua Doutor Plácido da Costa, 4200-450 Porto, Portugal
[9] Unit of Anatomy, Department of Biomedicine, Faculty of Medicine, University of Porto, Alameda Professor Hernâni Monteiro, 4200-319 Porto, Portugal
[10] Department of Community Medicine, Health Information and Decision (MEDCIDS), Faculty of Medicine, University of Porto, Rua Doutor Plácido da Costa, 4200-450 Porto, Portugal
[*] Correspondence: nunovale@med.up.pt; Tel.: +351-220-426-537.

Supplementary Material

Table S1. List of the most important abbreviations

Abbreviation	Meaning
CORT	Corticosterone
5-HIAA	5-hydroxyindoleacetic acid
5-HT	Serotonin
5-HTP	5-Hydroxytryptophan
H_2O_2	Hydrogen peroxide
L-Trp	L-Tryptophan
ROS	Reactive oxygen species
